# Circadian Activity Disruption in Cardiac Remodeling Patients Underlies Autonomic Dysfunction in Heart Failure

**DOI:** 10.3390/biomedicines14051054

**Published:** 2026-05-06

**Authors:** Natalia Buitrago-Ricaurte, Andre J. Riveros, Rafael González Niño, Liliana Otero, Juan David Meléndez, Alain Riveros-Rivera

**Affiliations:** 1 Escuela de Medicina y Ciencias de la Salud, Universidad del Rosario, Bogotá 111221, Colombia; 2 Departamento de Biología, Escuela de Ciencias e Ingeniería, Universidad del Rosario, Bogotá 111221, Colombia; andre.riveros@urosario.edu.co; 3 Department of Neuroscience, College of Science, University of Arizona, Tucson, AZ 85721, USA; 4 Servicio de Cardiología, Instituto del Corazón de Bucaramanga-Sede Bogotá, Bogotá 110311, Colombia; rafaelgonzaleznino@gmail.com (R.G.N.); mdavid0995@gmail.com (J.D.M.); 5 Centro de Investigaciones Odontológicas, Pontificia Universidad Javeriana, Bogotá 110231, Colombia; lotero@javeriana.edu.co; 6 Departamento de Ciencias Fisiológicas, Facultad de Medicina, Pontificia Universidad Javeriana, Bogotá 110231, Colombia; riveros-a@javeriana.edu.co

**Keywords:** circadian rhythm, heart failure, sex characteristics, aging, heart rate determination, autonomic nervous system

## Abstract

**Background:** Heart failure (HF) is a complex clinical syndrome that presents significant challenges in diagnosis and treatment. Exploring innovative pathways to better understand the physiopathological mechanisms has led to the concept of cardiac remodeling (CR), which helps elucidate the diversity in clinical manifestations and treatment responses. However, the extent to which CR influences autonomic cardiac dynamics across the circadian cycle remains unclear. **Methods:** Recordings of 24 h ECG recordings from 86 Control subjects and 86 patients meeting the criteria for cardiac remodeling were analyzed. Heart rate variability (HRV) parameters were estimated using 5 min Blackman–Harris windows per hour. Autonomic influences on cardiac electrical activity were assessed using time-domain, frequency-domain, and nonlinear methods. Circadian parameters (MESOR, amplitude, and acrophase) were derived via Cosinor modeling, and group differences were evaluated while controlling for age, sex, and medication effects. **Results:** Patients with CR exhibited reduced oscillatory activity in HRV measures compared with Control. MESOR and amplitude were significantly lower in CR patients, who also displayed an advanced-phase phenotype across multiple HRV domains over 24 h. Additionally, CR patients showed decreased complexity and entropy in nonlinear dynamics. **Conclusions:** Altered circadian rhythmicity of cardiac electrical activity is evidenced in cardiac remodeling by changes in circadian HRV parameters and nonlinear dynamics.

## 1. Introduction

Heart failure (HF) is a widespread syndrome driven by major cardiovascular diseases and risk factors, affecting millions globally, burdening healthcare systems, and deeply impacting individuals’ lives [1]. Key challenges to address HF include improving early detection, slowing disease progression and optimizing therapies. To meet these challenges, a unifying pathophysiological lens traces HF back to its structural and molecular roots, proposing an approach based on an integrated concept. Cardiac remodeling, the pathological alteration of cardiac structure and function, provides an integrative model encompassing immune, metabolic, autonomic, and electrical processes as part of a dynamic and interconnected continuum. Although relatively new, the concept of cardiac remodeling has emerged as a powerful response to the calls for comprehensive pathophysiological frameworks that facilitate the identification of novel biomarkers and therapeutic targets within heart failure syndrome [2].

Cardiac remodeling (CR) is a multifactorial phenomenon influenced by age and sex and encompassing classical cardiovascular risk factors [3]. Persistent hypertension, dyslipidemia, and structural abnormalities drive sustained immune, metabolic, and autonomic alterations that ultimately converge on remodeled myocardial tissue [4]. Moreover, the interplay of these factors with age and sex interferes on key biological processes (e.g., mitochondrial dysfunction, increased oxidative stress, impaired cellular repair, and sex hormone-dependent differences in immune response, myocardial fibrosis, and autonomic regulation), leading to a synergy that derives from a maladaptive state revealed via clinical manifestations and the progression of HF symptoms [5,6]. Nevertheless, heterogeneous phenotypes, diverse clinical presentations, and variable pharmacological responses to treatment highlight the need to investigate unexplored underlying factors that drive cardiac remodeling in HF.

We address this gap by considering circadian rhythm as the key mechanism that bridges underlying factors in CR and HF clinical manifestations [7,8]. Circadian clocks, understood as the molecular assemblies that orchestrate rhythmic protein expression, are essential for maintaining oscillatory patterns in cardiovascular function. For example, approximately 10–30% of the transcriptome in pacemaker cells and cardiomyocytes is regulated by peripheral clocks [9]. Unlike the central clock located in the Suprachiasmatic Nucleus (SCN), peripheral clocks are functional molecular oscillators in various tissues and organs that regulate 24 h rhythmicity across multiple physiological systems [10]. These peripheral clocks are entrained by inputs from the central pacemaker as well as local factors such as hormones, metabolites, and autonomic innervation [11,12]. In the context of CR, metabolic, immune, and autonomic disturbances of peripheral oscillators impair cardiovascular function as a secondary phenomenon following chronodisruption.

Understanding the influence of circadian rhythms in HF through CR is essential to elucidate the temporal patterns of pathophysiological processes that shape morbidity, mortality, and prognosis. However, despite growing interest, most research focuses on the central circadian clock, leaving aside significant gaps in our understanding of the contribution of cardiac oscillators to the pathophysiology, phenotypic variability, and therapeutic responsiveness in HF. Hence, we address the interplay between cardiac remodeling and autonomic circadian activity in HF patients who met established CR criteria. Specifically, we analyzed 24 h ECG recordings to estimate heart rate variability (HRV) parameters as proxies of autonomic activity. We characterized circadian dynamics and assessed the influence of age and sex, comparing the results with a matched cohort of healthy individuals.

## 2. Materials and Methods

### 2.1. Subjects and Data Collection

We evaluated data from 172 individuals (86 Control, 86 patients with cardiac remodeling) matched by sex (Table 1). Individuals in the Control group were obtained from the Telemetric and Holter ECG Warehouse database of the University of Rochester Medical Center [13]. We complemented this dataset with data from individuals in the remodeling group, which was collected at the Instituto del Corazón de Bucaramanga.

The individuals in the Control group complied with the following inclusion criteria:

1. No overt cardiovascular disease or history of cardiovascular disorders (including stroke, TIA, peripherical vascular disease).
2. No history of high blood pressure (>150/90).
3. No medication.
4. No chronic illness (e.g., diabetes, asthma, chronic obstructive pulmonary disease, etc.).
5. The subject was not enrolled if they were evaluated by a physician for cardiovascular-related syndrome (chest pain, palpitation, syncope) but was otherwise diagnosed as being healthy.
6. No abnormal physical examination.
7. Sinus rhythm in 12-lead ECG without any suspicious abnormalities (e.g., signs of ventricular hypertrophy, inverted T-wave, intraventricular conduction disturbances).
8. Normal echo and normal ECG exercise testing in presence of suspicious ECG changes.
9. No pregnancy.

Cardiac remodeling patients (CR) were included according to the following inclusion criteria:

1. History of cardiovascular diseases (hypertension, coronary artery disease, heart failure, stroke, TIA, peripherical vascular disease).
2. At least two of the established CR criteria [2]: altered ventricular mass (VM), augmented relative wall thickness (RPT), posterior or septal wall thickening, or increased LV mass index (see Table 1).
3. Complete clinical records.
4. No reported arrhythmia.
5. No pregnancy.

The recordings of the individuals in the Control group were acquired using the SpaceLab-Burdick digital Holter recorder (SpaceLab-Burdick, Inc., Deerfield, WI, USA). The cardiac remodeling recordings were acquired using the Sentinel-Pathfinder SL^®^ (Spacelabs healthcare, Inc., Issaquah, WA, USA; V.9800). To ensure comparability between datasets, acquisition and preprocessing procedures were harmonized across groups. For both groups, before starting ambulatory recording, the researchers allowed the subjects a supine resting period of 20 min. Then, 24 h ambulatory monitoring of the electrocardiogram activity was conducted using three leads with a pseudo-orthogonal configuration (X, Y, and Z) [14,15]. The sampling frequency was 200 Hz, and the amplitude resolution was 10 μV. The recordings were visually supervised and filtered manually for artifacts by a specialized technician using Vision Premier in Control (SpaceLab-Burdick, Inc., Deerfield, WI, USA) and using Sentinel-Pathfinder SL^®^ (Spacelabs healthcare, Inc., Issaquah, WA, USA; V.9800) for CR patients. This harmonized preprocessing approach aimed to minimize inter-system variability and ensure consistency in signal quality, artifact correction, and HRV analysis.

Because the Control dataset lacked detailed information on environmental light exposure or season of acquisition, these variables could not be matched or controlled between groups. For the CR group, recordings were obtained under standard ambulatory conditions without controlled light exposure, thereby reflecting routine real-world clinical practice.

Once we compiled the entire database, we selected recordings of at least 24 h and data quality according to the noise detection score (<1%) determined by the software Kubios (Kubios HRV Scientific, v4.2.0, Kubios Oy, Kuopio, Finland). Then, we conducted a retrospective observational study including 172 subjects [16]. We determined analysis groups: “Control,” and “CR”; we further classified individuals as “Young,” “Middle-aged,” and “Old” based on 30–50 years, 51–60 years, and 61–80 years, respectively. We also included the following variables for further analysis: sex, Body Mass Index (BMI), self-reported smoking, systolic and diastolic blood pressure.

### 2.2. Analysis of Heart Rate Variability

Preprocessing: We used preprocessing for the tools and visual inspection available in Kubios HRV Scientific v 4.2.0. Specifically, 5 min windows were selected from each hour, and the software’s functions for noise detection, beat correction, and segment selection were applied. This approach allowed us to identify arrhythmias and to correct beat misclassifications and artifacts, thereby improving the quality and reliability of the HRV analysis [17]. We excluded 15 Control and 19 CR recordings due to strong threshold beat correction (excluding >5% of total beats). Three analyzed recordings from Control required beat correction, with heartbeats corrected within 1% and 3%. Only segments classified as stationary on the basis of preprocessing were retained for analysis.

Analysis in the time domain: We identified each R peak and selected the intervals between two normal complexes (hereafter NN intervals) [17], based on a cubic spline interpolation filter with a maximal correction factor of 20% using Kubios HRV Scientific. We obtained four indices in the time domain: i. the standard deviation of all NN intervals (SDNN), ii. the standard deviation of the average NN intervals (SDANN), iii. Root Mean Square of the difference between successive NN intervals (RMSS), and iv. the proportion of adjacent normal NN intervals differing by more than 50 ms (pNN50).

Analysis in the frequency domain: We conducted a Fast Fourier Transform using a single preprocessed, artifact-free 5 min segment per hour. A Blackman–Harris window per hour was applied to minimize spectral leakage and enhance the reliability of power spectral estimates (spectral resolution: 300 points/Hz; interpolation rate: 4 Hz). Power spectral densities were obtained by integrating the power spectral density in each frequency band: high frequency (HFab: 0.15–0.5 Hz), Low Frequency (LF: 0.04–0.15 Hz), and Very Low Frequency (VLF: 0.0033–0.04 Hz) [18]. We also calculated and expressed the LF/HF index in a normalized power spectrum.

Analysis of nonlinear parameters: We relied on fractal and multifractal methods (detrended fluctuation analysis), graphical methods (Poincaré plot), and information methods (approximate entropy, sample entropy, Shannon entropy) [19,20]. Nonlinear HRV indices capture complementary aspects of heartbeat dynamics and autonomic regulation. Fractal and multifractal methods, including detrended fluctuation analysis, are used to assess the presence of short- and long-term correlation properties in RR intervals. Graphical methods based on the Poincaré plot are used to characterize beat-to-beat variability and the dispersion patterns of RR intervals. In addition, information-based metrics, including approximate entropy, sample entropy, and Shannon entropy, were used to quantify complexity, regularity, and uncertainty of heart rate dynamics [20].

Autonomic indexes: We analyzed three parameters derived from HRV metrics that reflect the influence of the parasympathetic and sympathetic branches of the autonomic nervous system. Each parameter’s value is scaled by the standard deviation of a normal population and presented in terms of these deviations [21]. Significant autonomic activity is defined as being above or below 1 SD from the norm for each case. The Parasympathetic Nervous System Index (PNSi) is calculated based on the mean RR interval, Root Mean Square of Successive Differences (RMSSD), and the Poincaré plot index SD1. The Sympathetic Nervous System Index (SNSi) is calculated based on the mean heart rate (HR), Baevsky’s Stress Index [22], and the Poincaré plot index SD2. The Stress Index (STi) is the square root of Baevsky’s Stress Index.

### 2.3. Analysis of Circadian Rhythmicity

We analyzed whether heart rate variability (HRV) parameters followed a circadian pattern over the 24 h sampling interval. First, we tested uniformity and oscillation using repeated measures ANOVA (see Supplementary Table S1). After rejecting uniformity, we applied the Cosinor fitting method to calculate the MESOR, amplitude, and acrophase (ϕ) (Equation (1)). To analyze acrophase dispersion and tendency, we applied the Rayleigh test. We further evaluated potential differences in these variables between groups (Control vs. CR).

(y = M + Acos + ϕ),(1)

### 2.4. Statistical Analyses

We calculated descriptive statistics for all the variables studied. Continuous variables are presented as means or medians, whereas categorical variables (sex, race, and smoking) are described as absolute frequencies and compared between groups (Control, CR) using a chi-squared test. A total of 19 HRV variables were analyzed: three from the time domain, four from the frequency domain, nine corresponding to nonlinear, and three from autonomic indexes.

Rhythmicity analysis began with a uniformity test to exclude non-rhythmic patterns, followed by cross-validation between the repeated measures ANOVA and the Fourier-based non-negative least squares algorithm (FFT-NNLS) [23]. Once rhythmicity was confirmed, a Cosinor model was fitted to estimate the circadian parameters MESOR, amplitude, and acrophase (ϕ). The Rayleigh test was applied to assess phase distribution.

Comparisons of Cosinor-derived parameters between groups were performed using the Mann–Whitney U test after assessing data distribution with the Kolmogorov–Smirnov test. Because of multiple comparisons in non-parametric hypothesis testing, the Cliff method was used to adjust *p*-values in the post hoc analysis. Given the large number of comparisons across HRV indices and circadian parameters, *p*-values were further adjusted for multiple testing using the false discovery rate approach according to the Benjamini–Hochberg procedure. Thus, the statistical pipeline for this analysis was: 1. initial non-parametric group comparisons with the Mann–Whitney U test; 2. estimation of effect size using Cliff’s delta; and 3. FDR correction for multiple comparisons.

Finally, to evaluate the effect of age, sex, beta-blocker use, and CR on Cosinor parameters, we performed a three-way ANOVA with a 95% Confidence Interval. We performed statistical tests using the card package in R Statistical software (v 4.1.2; R Core Team 2021) and the software JMP^®^ (v. 18; SAS Institute Inc., Cary, NC, USA). Statistical significance was considered at *p* < 0.05.

### 2.5. Ethical Considerations and Approval

This study received approval from the Ethical Committee of the Instituto del Corazón de Bucaramanga, Sede Bogotá, Colombia (protocol number 06, 8 November 2023).

## 3. Results

### 3.1. Subjects

**Figure 1:**
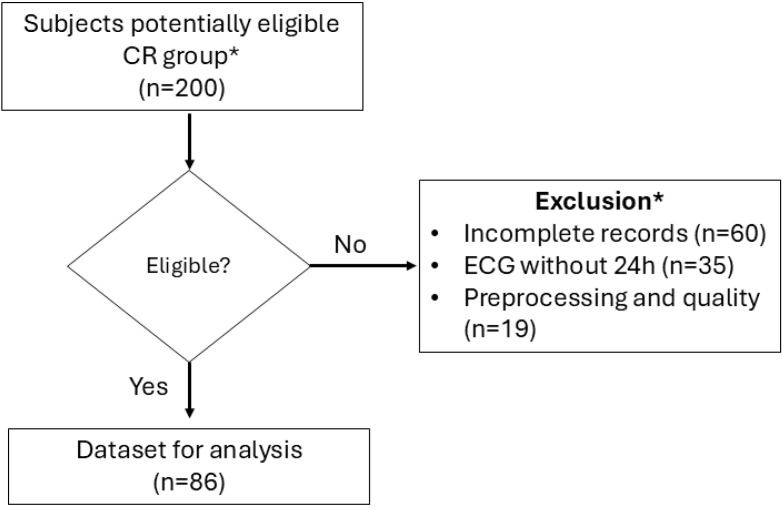
Flowchart of patient selection. * Control group participants were matched to cases by sex.

We analyzed 172 subjects (Control = 86, CR = 86; Figure 1). We found that none of the 24 variables analyzed followed a normal distribution (Kolmogorov–Smirnov test, analyses for normality tests). We only found differences between median age, self-reported smoking, and hypertension (see Table 2 for results across all variables). Eighty-six patients met at least two echocardiographic criteria for cardiac remodeling and were distributed by ejection fraction as follows: HFrEF: 18–21%; HFimpEF: 10–11.6%; and HFpEF: 58–67.4% (see Supplementary Table S2). Forty-two patients were classified as class III according to the New York Heart Association (NYHA) severity classification.

### 3.2. Analysis of Circadian Rhythmicity in CR

Before Cosinor analysis, rhythmicity was screened using complementary criteria rather than relying on a single test. First, repeated measures ANOVA was used as an initial screening tool to identify variables showing significant time-of-day variation across the 24 h cycle. Variables that did not show significant temporal variation were considered not to meet the minimum criterion for circadian rhythmicity and were not prioritized for Cosinor interpretation. We found uniformity in the distribution of data across five parameters within the CR subjects (one-way repeated measures ANOVA: VLF: F_23,86_ = 0.86, *p* = 0.649; SD2/SD1: F_23,86_ = 1.00, *p* = 0.457; SamPEN: F_23,86_ = 1.32, *p* = 0.14; Alf1: F_23,86_ = 1.14, *p* = 0.289; DET: F_23,86_ = 1.45, *p* = 0.07). In the subjects within the Control group, only ShannE exhibited uniformity in the distribution (one-way repeated measures ANOVA: F_23,86_ = 1.21, *p* = 0.223). Second, rhythmicity was cross-validated using visual inspection of the 24 h profiles of the oscillatory patterns in the spectrograms. After these screening steps, Cosinor analysis was applied to variables meeting the rhythm-detection criteria, and model fit was evaluated based on the statistical significance of the fitted rhythm. Phase clustering was assessed using the Rayleigh test. This combined approach was used to provide a more robust and standardized basis for rhythm detection before proceeding with Cosinor modeling (see Supplementary Table S1).

We estimated the MESOR, amplitude, and acrophase (Φ) for 18 heart rate variability (HRV) parameters using a Cosinor fitting method (Table 3).

**Figure 2:**
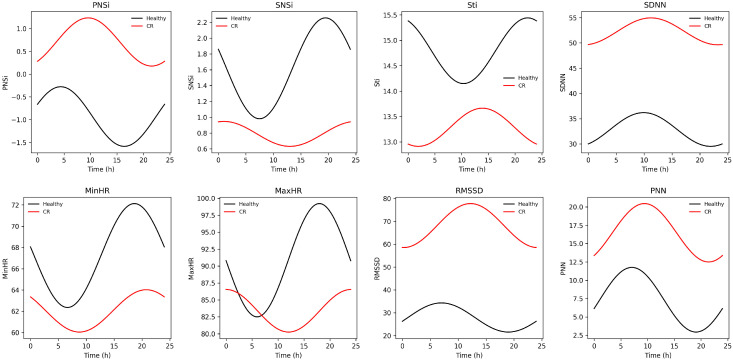
Variation in HRV time-domain parameters between groups across 24 h. CR patients exhibited an overall shift toward altered autonomic balance. The sympathetic index (SNSi) and Stress Index (STi) displayed reduced oscillatory amplitude and an earlier acrophase compared with healthy subjects, indicating attenuated rhythmic sympathetic modulation. In contrast, the Parasympathetic Index (PNSi), SDNN, RMSSD, and pNN50 (PNN) demonstrated higher overall levels in CR subjects with preserved phase alignment relative to healthy individuals. Regarding heart rate dynamics, both minimum (MinHR) and maximum heart rate (MaxHR) exhibited reduced oscillatory amplitude in CR patients. Notably, MaxHR showed an earlier acrophase, indicating a phase advance in peak cardiac activation. These findings indicate diminished rhythmic autonomic modulation with selective phase alterations in CR.

- Time-domain HRV parameters:

We observed significant differences between groups in MESOR and amplitude (Table 2). Notably, CR subjects exhibited an advanced acrophase in parasympathetic-related variables assessed by the Watson–Williams circular test: RMSSD Φ (median-IQR): Control: 3:25-11 h, CR: 00:23-8 h; *p* = 0.003 and pNN50 Φ (median-IQR): Control: 3:18-10 h, CR: 1:05-10 h; *p* = 0.002 (see Supplementary Table S4). Although SDNN, RMSSD, and pNN50 appeared higher in CR subjects, these indices are highly susceptible to the presence of non-NN intervals, reflecting increased rhythm irregularity rather than enhanced variability. In our population, a higher arrhythmogenic burden was frequently observed in HF subjects compared with Control, likely secondary to structural, metabolic, and electrical remodeling. A sub-analysis excluding 11 individuals with more than 10% non-NN intervals revealed that time-domain activity was consistently lower in CR patients (see Supplementary Table S4), confirming a reduction in overall HRV activity (Figure 2).

The autonomic indexes showed that PNSi is higher in CR subjects (MESOR (median-IQR): Control: −1.09–1.1, CR: 0.42–2.9; *p* < 0.001) and exhibits an advanced-phase PNSi Φ (median-IQR): Control: 04:46-2 h, CR: 02:38-7 h; *p* < 0.001. The sympathetic-related variables Stress Index and SNS were lower in CR subjects (SNS MESOR (median-IQR): Control: 1.5–1.5, CR: 0.2–1.7; *p* < 0.001); Stress Index MESOR (median-IQR): Control: 13.7 ± 7.2, CR: 10.6–7.4; *p* < 0.001.

- Frequency-domain HRV parameters:

We also found group differences in MESOR and amplitude for HFn, LFn, and LF/HF. Similar to the time-domain findings, CR subjects displayed an advanced acrophase in HFn: HFn Φ (median-IQR): Control: 04:34-10 h, CR: 01:03-10 h; *p* = 0.002. LF/HF MESOR was lower in CR subjects: LF/HF (median-IQR): Control: 4.11–3.1, CR: 1.6–2.6; *p* < 0.001.

**Figure 3:**
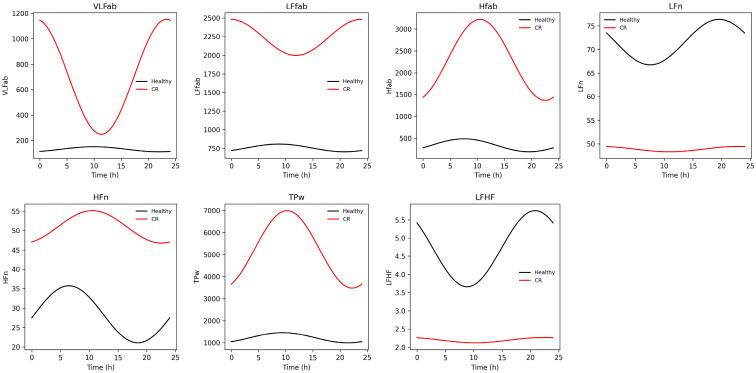
Variation in HRV frequency-domain parameters between groups across 24 h. The CR group demonstrated markedly reduced oscillatory amplitude in normalized Low Frequency (LFn) and the LF/HF ratio (LFHF), indicating attenuated sympathovagal balance rhythmicity. Conversely, Absolute Low Frequency (LFab), Absolute High Frequency (HFab), Normalized High Frequency (HFn), Very Low Frequency (VLFab), and total power (Tpw) exhibited greater oscillatory amplitude in CR patients. The exaggerated oscillations in absolute spectral components suggest increased total variance but altered proportional distribution across bands. While absolute components increased, normalized indexes and LF/HF were reduced, indicating that relative spectral balance differs despite higher total power oscillation. CR is characterized by spectral redistribution and altered rhythmic coordination rather than uniform suppression of variability.

While HFab is a vagal-related parameter, when we analyzed the normalized index (HFab/HFab + LFab), we found that CR subjects exhibited a higher relative HF contribution compared with Control (Controls 0.13; CR: 0.25). Probably, that apparent increase in normalized HFab in CR is not due to enhanced vagal activity, but rather reflects a disproportionate reduction in LF and total power, consistent with a global loss of autonomic oscillatory power (Figure 3).

**Figure 4:**
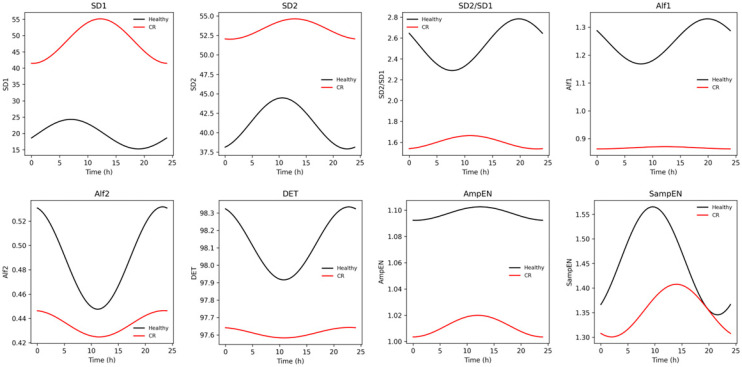
Variation in HRV nonlinear parameters between groups across 24 h. Nonlinear metrics provide a more sensitive characterization of altered rhythmic complexity. CR patients showed reduced oscillatory amplitude across multiple nonlinear domains: graphical (lower SD2/SD1 ratio), fractal (reduced α1 and α2), entropy-based (reduced AmpEn and SampEn), and Recurrence Determinism (reduced DET). Although some metrics exhibited preserved general rhythmic patterns, the amplitude of oscillation was consistently attenuated in CR. This indicates reduced dynamic adaptability and diminished multiscale complexity.

- Nonlinear HRV parameters:

We found significant differences in MESOR and amplitude for SD1, SD2, AmpEN, Alf2, CorrDim, and REC (Table 2). Specifically, we observed that CR patients exhibited higher SD1 MESOR (median-IQR: Control: 16.73–14.12 ms, CR: 31.14–81.1 ms; *p* = 0.001) and lower SD2 MESOR (median-IQR: Control: 39.89–22.3 ms, CR: 49.12–49.2 ms; *p* < 0.001), AmpEN MESOR (median-IQR: Control: 1.1–0.08 ms, CR: 1.06–0.2 ms; *p* < 0.001), Alf2 MESOR (median-IQR: Control: 0.48–0.12, CR: 0.41–0.19; *p* < 0.001), CorrDim MESOR (median-IQR: Control: 1.02–1.6, CR: 0.4–0.19; *p* < 0.001) and REC MESOR (median-IQR: Control: 34.4–5.5, CR: 35.23–17.5; *p* = 0.03). Amplitude differences were proportionally correlated with MESOR for each variable. SD1 and SD2 were higher in CR; however, the SD2/SD1 ratio was significantly reduced, indicating impaired long-range oscillatory dynamics and diminished regulatory integration, likely related to altered autonomic balance, reduced baroreflex engagement, and disrupted multiscale control mechanisms. In contrast, entropy-based measures demonstrated greater discriminatory capacity, exhibiting a consistent pattern of reduced MESOR and amplitude, reflecting attenuated oscillatory activity and loss of complexity in CR subjects (Figure 4).

### 3.3. Age and Sex Effect on Circadian Parameters

We found significant effects of cardiac remodeling across all variables in the analysis of the circadian parameters related to time-domain variables. Age and remodeling were associated with an advanced acrophase in the SNS Index (mean phase: Older CR = 17:07 ± 0.15 h; Older Control = 19:47 ± 0.24 h; ANOVA: age: F_2,172_ = 5.76, *p* = 0.01; remodeling: F_2,172_ = 3.44, *p* = 0.03), SDNN (mean phase: Older CR = 17:10 ± 0.38 h; Older Control = 00:21 ± 0.24 h; ANOVA: age: F_2,172_ = 3.54, *p* = 0.03; remodeling: F_2,172_ = 9.3, *p* < 0.0001), RMSSD (mean phase: Older CR = 17:47 ± 0.39 h; Older Control = 23:17 ± 0.25 h; ANOVA: age: F_2,172_ = 3.43, *p* = 0.03; remodeling: F_2,172_ = 9.02, *p* = 0.0002), and delayed phase in the pNN50 (mean phase: Older CR = 23:12 ± 0.27 h; Older Control = 18:58 ± 0.42 h; ANOVA: age: F_2,172_ = 3.7, *p* = 0.02; remodeling: F_2,172_ = 5.03, *p* = 0.007). Also, age and sex were significantly associated with differences in amplitude (mean phase: Older CR = 6.61 ± 0.85; Older Control = 2.27 ± 1.3 h; ANOVA: age: F_2,172_ = 3.3, *p* = 0.02; remodeling: F_2,172_ = 6.17, *p* = 0.01).

Similarly, we found significant effects of age on frequency-domain parameters. Older CR subjects (n = 30) exhibited lower low-frequency activity (LF MESOR and Amplitude) over 24 h and higher high-frequency activity than Older Control (HFab MESOR). Older Control subjects exhibited higher LF MESOR than Older CR (Older CR: 41.31 ± 2.33; Older Control: 63.14 ± 3.67 age: F_2,172_ = 9.65, *p* < 0.0001; remodeling: F_2,172_ = 61.32, *p* < 0.001). In contrast, Older Control subjects exhibited lower HFab MESOR than Older CR (Older CR: 58.4 ± 2.31; Older Control 36.74 ± 3.6; age: F_2,172_ = 9.64, *p* < 0.0001; remodeling: F_2,172_ = 61.50, *p* < 0.001).

Interestingly, we found an effect of sex on LF; females exhibited lower amplitude than Control women (CR female: 6.97 ± 0.84 vs. Control female: 10.18 ± 0.82; ANOVA: F_2,172_ = 4.47, *p* = 0.03; remodeling: F_2,172_ = 61.32, *p* < 0.001). Moreover, female CR exhibited lower amplitude compared to female in the Control group (CR: 6.9 ± 0.84; Controls: 10.17 ± 0.82; ANOVA: sex * group: F_2,172_ = 4.4, *p* = 0.03). Lastly, age, sex, and group were associated with differences in LF/HF MESOR (age: F_2,172_ = 6.61, *p* = 0.001; sex: F_2,172_ = 4.11, *p* = 0.04; remodeling: F_2,172_ = 39.21, *p* < 0.001). Older CR female exhibited lower MESOR than older female in the Control group (Older CR female: 2.25 ± 0.34; Older Control female: 3.87 ± 0.33).

Finally, we found further evidence of lower sympathetic activity and delayed parasympathetic activity and phase in CR patients when analyzing the nonlinear time series parameters. Age and remodeling were significantly associated with an advanced phase of the SD1 acrophase (Older CR: 23:17 ± 0.25 h; Older Control: 17:47 ± 0.3 h; age: three-way ANOVA: F_2,172_ = 3.44, *p* = 0.03; age * group: three-way ANOVA: F_2,172_ = 9.02, *p* = 0.0002). Moreover, we found a significantly lower SD2/SD1 MESOR (Older CR: 1.3 ± 0.09; Older Control 2.05 ± 0.14; age: three-way ANOVA: F_2,172_ = 10.92, *p* < 0.0001; group: three-way ANOVA: F_2,172_ = 61.4, *p* < 0.0001) and amplitude in Older CR subjects (Older CR: 0.19 ± 0.03; Older Control: 0.28 ± 0.05; age: three-way ANOVA: F_2,172_ = 8.86, *p* = 0.0002; group: three-way ANOVA: F_2,172_ = 12.5, *p* = 0.0005). Also, the acrophase of SD2/SD1 was significantly affected by the interaction of age and sex; middle-aged females exhibited an advanced phase (older female: −3.53 ± 0.29; younger female: −2.36 ± 0.35; age * sex: three-way ANOVA: F_2,172_ = 3.79, *p* = 0.02).

We also found a significant effect of sex and remodeling on the entropy measures: the AmpEN MESOR, amplitude, and acrophase, and the SampEN MESOR. On the one hand, females in the CR group exhibited lower AmpEN MESOR (CR female: 1.02 ± 0.02; Control female: 1.12 ± 0.02; sex: three-way ANOVA: F_2,172_ = 4.48, *p* = 0.03; group: three-way ANOVA: F_2,172_ = 14.11, *p* = 0.0002), lower amplitude (female: 0.06 ± 0.004 vs. male: 0.06 ± 0.004; sex: three-way ANOVA: F_2,172_ = 4.96, *p* = 0.02) and an advanced phase (female: 18:37 ± 0.18; male: 19:00 ± 0.11; sex: three-way ANOVA: F F_2,172_ = 5.6, *p* = 0.01). On the other hand, sex, but not remodeling, significantly affected SampEN, such that females exhibited a higher MESOR independently of their remodeling condition (female: 1.45 ± 0.03 vs. male: 1.33 ± 0.03; sex: three-way ANOVA: F_2,172_ = 5.23, *p* = 0.02).

To understand the influence of beta-blocker use on circadian cardiac dynamics, we estimated Cosinor parameters for each subject and variable, then fit a multivariable model. In our cohort, thirty-three CR subjects (38.4%) were receiving beta-blocker therapy, while no use was reported among healthy subjects. Across the whole cohort, linear regression models adjusted for group, age, and sex showed that beta-blocker use exerted a main effect on MESOR and, to a lesser extent, on amplitude for a subset of autonomic and nonlinear indices (Table 4). Importantly, CR group-related effects remained significant after adjustment, indicating that the observed circadian alterations were not solely related to pharmacological treatment. In CR sub-analysis, beta-blocker use was associated with a modest effect on circadian parameters: SDNN MESOR (β = −26.1, *p* = 0.04, q = 0.18); pNN50 MESOR (β = −14.8, *p* = 0.004, q = 0.10); REC Amplitude (β = 2.42, *p* = 0.02, q = 0.56); REC MESOR (β = 7.68, *p* = 0.01, q = 0.11); ShannE MESOR (β = 0.20, *p* = 0.01, q = 0.10); SD2 MESOR (β = −23.9, *p* = 0.03, q = 0.19) whereas age and sex contributed differently, as described previously. Acrophase analyses using the Watson–Williams test revealed no systematic phase reorganization secondary to beta-blocker use. No significant differences in HRV parameters were observed between the self-reported smokers and non-smokers.

## 4. Discussion

Heart failure is a complex clinical syndrome closely linked to cardiac remodeling (CR), a process marked by structural and functional alterations. In this study, we investigated how cardiac remodeling (CR) affects cardiac autonomic activity across the circadian cycle. Overall, we observed that CR is associated with a chronodisrupted phenotype. Individuals with CR exhibited reduced short- and long-term autonomic activity, decreased entropy and system complexity, and a phase-shifted circadian pattern. These findings suggest that cardiac remodeling is accompanied by impaired circadian organization of autonomic function rather than a simple reduction in heart rate variability magnitude.

CR patients demonstrated an altered spectral profile. Specifically, higher total power and higher Absolute and Normalized High-Frequency components were observed together with a lower LF/HF ratio. This pattern does not indicate global enhancement of autonomic modulation; instead, it reflects a redistribution of oscillatory power toward faster, respiration-locked components with attenuation of slower regulatory dynamics. Such redistribution may represent a loss of multiscale autonomic integration rather than increased vagal efficiency. We considered three possible explanations for this unexpected profile: methodological artifacts, medication effects, and disease-related alterations of oscillatory structure. Signal preprocessing was carefully controlled using a combination of manual and automated routines, and individuals with more than 10% non-NN intervals were separately evaluated. Moreover, additional analysis showed that beta-blocker use did not significantly affect frequency-domain parameters. Thus, the observed pattern appears more consistent with CR-related autonomic remodeling characterized by impaired baroreflex–autonomic integration and respiration-locked variability [24,25].

Nonlinear dynamics further supported the presence of altered autonomic organization in CR patients. We observed significant differences in MESOR and amplitude across graphical, entropy-based, and fractal nonlinear metrics. CR patients exhibited short-term variability components that were relatively preserved or increased, but long-range and oscillatory structure, as well as integrated regulatory control, were attenuated. A reduced SD2/SD1 ratio has been interpreted as impaired long-term autonomic coordination, potentially reflecting diminished baroreflex contribution and reduced multiscale integration of cardiovascular regulation [26,27]. Likewise, the reduction in entropy and correlation dimension indicates decreased signal irregularity and lower dynamical richness, consistent with a loss of physiological complexity. Together, these nonlinear findings indicate that CR is not only associated with reduced variability magnitude but also with a structural simplification of heart rate dynamics. In this context, the tendency we observed toward a circadian phase shift in CR should be interpreted with caution, since multiple external factors, including light exposure, sleep, medications, and physical activity, may influence circadian timing. These confounders are known to affect circadian phase particularly strongly, as phase is the most sensitive shiftable dimension of the circadian system in response to environmental cues [28]. Therefore, the chronodisruption phenotype in CR likely reflects a global attenuation of oscillatory adaptability rather than an isolated phase alteration or a shift in single parameters.

The chronodisrupted phenotype observed in CR may be explained by converging inflammatory, metabolic, and autonomic alterations that affect peripheral oscillators. Cardiac remodeling involves immune cell recruitment and sustained expression of proinflammatory mediators, a process referred to as immune remodeling [29,30]. Replacement of contractile myocardium with fibrotic tissue represents the final stage of chronic structural and metabolic stress and has been associated with altered expression of core clock genes [8,31]. Remodeled cardiac tissue exhibits a distinct inflammatory profile characterized by elevated levels of proinflammatory cytokines (TNF-α, IL-6, IL-1β, TGF-β), sustained M1 macrophage polarization, Th1-skewed immune responses, and persistent fibroblast activation [29,30,32]. Notably, TNF-α and IL-1β suppress the expression of circadian genes such as CLOCK, BMAL1, and PER1 through NF–κB-mediated transcriptional repression [33,34]. Downregulation of REV-ERBα further reinforces inflammatory signaling, establishing a feedback loop between inflammation and circadian disruption. These mechanisms provide biological plausibility for the phase shifts and amplitude reductions identified in our cohort [33,35,36].

Metabolic remodeling represents another intersecting pathway. CR is associated with reduced fatty acid oxidation, impaired glucose metabolism, and increased reliance on alternative substrates, accompanied by elevated oxidative stress [37,38]. Redox imbalance modulates CLOCK and BMAL1 activity and influences post-translational modifications of clock proteins [39,40,41]. Signaling pathways involving NRF2, SIRT1, NAD^+^ metabolism, and AMPK link metabolic state to circadian gene regulation at transcriptional and epigenetic levels [42,43]. These metabolic alterations intersect with neurohumoral activation and mitochondrial dysfunction, amplifying structural and functional maladaptation [7,44]. In particular, mitochondrial dysfunction plays a pivotal role in cardiac remodeling and chronodisruption [37]. The reduced energetic reserve observed in heart failure may be associated with alterations in clock gene expression, impaired oxidative phosphorylation, and increased oxidative stress, ultimately leading to disrupted cellular energetics and loss of circadian coordination. As a central mechanism linking metabolic, inflammatory, and circadian alterations, mitochondrial-related biomarkers such as mitochondrial DNA copy number, reactive oxygen species (ROS) levels, ATP production, and circulating markers of mitochondrial damage may have a promising role in heart failure detection and stratification. Therefore, the advanced-phase circadian phenotype observed in our patients may reflect disturbances in metabolic–circadian coupling, as supported by altered HRV rhythmicity across the 24 h cycle.

Autonomic remodeling further contributes to circadian misalignment [45]. Chronic hemodynamic overload, persistent inflammation, and neurohumoral activation promote sympathetic overactivity, renin–angiotensin–aldosterone system activation, and endothelial dysfunction [46,47,48]. Such alterations modify central autonomic setpoints and interfere with the coordination between afferent input, central processing, and efferent autonomic output [49,50]. Disruption of this integration may impair synchronization between central and peripheral oscillators [9,51,52]. Reduced adaptability to autonomic challenges and diminished physiological resilience, as reflected in decreased HRV complexity metrics, are consistent with this maladaptive state. Our findings are partially concordant with previous studies reporting reduced HRV and impaired autonomic regulation in heart failure populations [53]. However, unlike studies describing uniform sympathetic predominance, we observed a redistribution of spectral power rather than simple sympathetic dominance. This discrepancy may reflect differences in cohort characteristics, disease stage, or analytical approaches, suggesting that CR-related autonomic alterations are more heterogeneous than previously assumed.

The observed pattern could be explained by aging and the bidirectional influence between autonomic activity and peripheral clocks. First, autonomic aging is associated with reduced flexibility, robustness, and performance of heart electrical activity which conduces to chronodisruption [54,55,56]. Older individuals exhibited reduced oscillations, diminished dynamism, and decreased complexity of autonomic parameters, which likely contribute to lower physiological adaptability and increased vulnerability to circadian dysregulation [57,58,59]. Second, HF patients exhibit diminished HRV activity as the standard measures reported in the MyoVasc cohort [53]. The altered sympathovagal balance observed in CR patients could impact clock gene expression, disrupting its activity and manifesting as a chronodisrupted phenotype [60,61]. Together, these findings highlight the need for further studies with larger populations and experiments to elucidate vagal behavior in CR and its influence on circadian heart activity.

This study has several limitations. First, the sample size was moderate, which may limit generalizability. Second, heart failure is a complex and heterogeneous syndrome that should ideally be approached from a phenotypic perspective, integrating inflammatory status, clinical manifestations, and functional evaluation. In our cohort, nearly 70% of patients were classified as HFpEF based on echocardiographic findings; however, we recognize that, from a chronobiological perspective, heart failure should be understood more broadly and with greater phenotypic resolution based on inflammatory status, clinical manifestations, and functional evaluation. Third, although signal preprocessing was rigorously controlled and medication effects were evaluated, alterations in intrinsic electrical activity are well-recognized features of cardiac remodeling and likely contribute to the observed autonomic profile. In addition, hormonal influences should be considered in chronobiological research, and data should ideally be analyzed according to hormonal cycle phase. Finally, the cross-sectional design precludes causal inference regarding the directionality between remodeling processes and circadian disruption. Nevertheless, the integration of time-domain, frequency-domain, and nonlinear analyses across the circadian cycle strengthens the robustness of the findings and provides a multidimensional characterization of autonomic chronodisruption in cardiac remodeling.

Taken together, our results indicate that cardiac remodeling is associated with impaired circadian autonomic organization, reduced multiscale complexity, and redistribution of oscillatory dynamics [45,62,63]. These alterations may reflect interactions among inflammatory, metabolic, and autonomic remodeling processes that disrupt peripheral clock regulation. Further longitudinal and mechanistic studies are warranted to clarify whether restoration of circadian alignment could represent a therapeutic target in patients with cardiac remodeling.

## 5. Conclusions

Our results suggest that CR is associated with altered circadian autonomic cardiac activity through immune, metabolic, and autonomic adaptations. Individuals with CR exhibited signs of chronodisruption, reflected in diminished circadian dynamics of heart rate variability parameters across short and long timeframes. Specifically, CR subjects exhibited decreased entropy and complexity, increased recurrence in circadian cardiac dynamics, and a shift toward an advanced-phase circadian phenotype. Altogether, our results highlight the role of cardiac remodeling in impairing peripheral oscillator activity and support the notion that circadian alterations in cardiac autonomic dynamics may serve as biomarkers and contributors to cardiovascular dysfunction. This phenomenon may lead to increased susceptibility to arrhythmias, reduced cardiac efficiency, and heightened vulnerability to cardiovascular events.

## Figures and Tables

**Table 1:** Cardiac remodeling criteria.

Parameter	Value		Interpretation
Left Ventricle Parameters			
Ventricular Mass (VM)	Female >95 g/m^2^	Male >115 g/m^2^	Normal geometry: VM and RWT below reference values Concentric remodeling: normal VM, ↑RWT Concentric hypertrophy: ↑VM and ↑RWT Eccentric hypertrophy: ↑VM, normal RWT
Relative Wall Thickness (RWT)	>0.42		
Posterior Wall Thickness (cm)	0.6–0.9	0.6–1.0	Reference value
	1.0–1.2	1.1–1.3	Mildly abnormal
	1.3–1.5	1.4–1.6	Moderately abnormal
	≥1.6	≥1.7	Severely abnormal
LV Mass Index (g/m^2^)	44–88	50–102	Reference value
	89–100	103–116	Mildly abnormal
	101–112	117–130	Moderately abnormal
	≥113	≥131	Severely abnormal
Diastolic Volume (mL/m^2^)	35–75		Reference value
	76–86		Mildly abnormal
	87–96		Moderately abnormal
	≥97		Severely abnormal
Systolic Volume (mL/m^2^)	12–30		Reference value
	31–36		Mildly abnormal
	37–42		Moderately abnormal
	≥43		Severely abnormal
Ejection Fraction (%)	≥55		Reference value
	45–54		Mildly abnormal
	30–44		Moderately abnormal
	<30		Severely abnormal
Left Atrium Parameters			
Anteroposterior Diameter	Female	Male	
	2.7–3.8 cm	3–4 cm	Reference value
	3.9–4.2	4.1–4.6	Mildly abnormal
	4.3–4.6	4.7–5.2	Moderately abnormal
	≥4.7	≥5.2	Severely abnormal
Diameter Indexed to BSA	1.5–2.3		Reference value
	2.4–2.6		Mildly abnormal
	2.7–2.9		Moderately abnormal
	≥3 cm/m^2^		Severely abnormal
Left Atrial Area	≤20		Reference value
	20–30		Mildly abnormal
	30–40		Moderately abnormal
	>40		Severely abnormal
Indexed LA Volume	22 ± 6		Reference value
	29–33		Mildly abnormal
	34–39		Moderately abnormal
	≥40		Severely abnormal

Cardiac remodeling definitions are established based on echocardiographic guidelines. CR patterns were classified according to left ventricular mass index (LVMI) and relative wall thickness (RWT). LVMI was indexed to body surface area (BSA). Increased RWT was defined as >0.42. Remodeling patterns were categorized as follows: normal geometry (normal LVMI and RWT), concentric remodeling (normal LVMI with increased RWT), concentric hypertrophy (increased LVMI and RWT), and eccentric hypertrophy (increased LVMI with normal RWT). In our study, all echocardiographic measurements were performed by the same expert operator to reduce inter-operator variability.

**Table 2:** Baseline characteristics between Control and CR subjects.

	Control (*n* = 86)	CR (*n* = 86)	*p*
Age (years: median-IQR)	49–15	58–17	0.001 ^£^
Females (*n*-%)	41–47.6%	38–32.7%	0.819 ^Ş^
White ethnicity (*n*-%)	71–82.5%	70–81.3%	0.998 ^Ş^
BMI (median-IQR)	24.7–4.47	24.22–2.32	0.96 ^£^
Self-reported smokers (*n*-%)	34–39.5%	10–11.6%	0.005 ^Ş^
Hypertension (*n*-%)	5–4.3%	43–50%	<0.001 ^Ş^

^Ş^ χ2 = 0.372, df = 1. SD = Standard deviation. ^£^ Mann–Whitney test: U = 1720, *p* = 0.005, r = 0.32. IQR = Inter Quartile Range.

**Table 3:** Comparisons of MESOR, amplitude, and acrophase between healthy and cardiac remodeling patients for parameters of HRV in the time and frequency domains, the nonlinear parameters, and the autonomic indexes.

	Variable	MESOR		Amplitude		Acrophase (hh:mm)	
Time domain		**Control**	**CR**	**Control**	**CR**	**Control**	**CR**
	SDNN (ms)	33.7 [16]	43.03 [63] ^a^	5.7 [7]	8.1 [15] ^b^	01:11 [9]	01:13 [7]
	RMSSD (ms)	23.6 [19.9]	43.98 [104.6] ^a^	7.2 [8.8]	12.1 [29.1] ^a^	03:25 [11]	00:23 [8]
	pNN50 (%)	4.6 [9.3]	10.4 [25.4] ^a^	4.6 [6.6]	5.4 [6.5]	03:18 [10]	01:05 [10] ^c^
Frequency domain	HFn (%)	26.61 [14.17]	52.25 [33.04] ^a^	8.33 [8]	6.6 [5.8] ^b^	04:34 [10]	01:03 [10] ^c^
	LFn (%)	73.3 [14.14]	47.65 [33.22] ^a^	8.3 [8]	6.6 [5.9] ^b^	17:56 [4]	17:42 [9]
	VLF (ms^2^)	123.3 [78.9]	121.55 [2939]	52.1 [60.54]	949.7 [5848]	02:48 [7]	01:06 [7]
	Hfab (ms^2^)	220.8 [295.2]	537.1 [819]	81.5 [231]	109.3 [463]	04:23 [4]	01:20 [6]
	Lfab (ms^2^)	589.6 [665.8]	480.5 [706.1]	67.3 [360.3]	45.4 [403]	17:20 [4]	17:41 [6]
	LF/HF	4.11 [3.1]	1.6 [2.6] ^a^	1.6 [1.7]	0.53 [1] ^a^	17:58 [4]	18:10 [10]
Nonlinear	SD1 (ms)	16.73 [14.12]	31.14 [81.1] ^a^	5.1 [6.2]	8.56 [11.12] ^a^	03:25 [11]	00:23 [8]
	SD2 (ms)	39.89 [22.3]	49.12 [49.2] ^a^	6.7 [6.9]	9.4 [10.3] ^c^	23:59 [10]	00:13 [7]
	SD2/SD1	2.5 [0.8]	1.5 [1] ^a^	0.4 [0.4]	0.2 [0.2] ^a^	18:30 [4]	19:35 [12]
	AmpEN	1.1 [0.08]	1.06 [0.2] ^a^	0.05 [0.03]	0.06 [0.05]	16:01 [9]	18:47 [8] ^c^
	SampEN	1.46 [0.22]	1.42 [0.62]	0.14 [0.13]	0.15 [0.11]	03:09 [12]	20:25 [13] ^c^
	Alf1	1.3 [0.2]	0.82 [0.4] ^a^	0.16 [0.1]	0.11 [0.001] ^a^	17:35 [3]	20:09 [9]
	Alf2	0.48 [0.12]	0.4 [0.19] ^a^	0.07 [0.03]	0.06 [0.04] ^b^	18:21 [4]	18:50 [6]
	CorrDim	1.02 [1.6]	0.86 [0.9] ^c^	0.5 [0.4]	0.4 [0.4]	01:50 [11]	23:29 [10]
	REC (%)	34.34 [5.5]	35.23 [17.5]	4.2 [3.2]	6.02 [5.5] ^a^	18:27 [7]	18:14 [7]
	DET (%)	98.1 [0.9]	97.6 [1.95] ^a^	0.6 [0.4]	0.6 [0.6]	18:01 [5]	18:28 [10]
	ShannonE	3.27 [0.22]	3.18 [0.3]	0.13 [0.1]	0.2 [0.13]^c^	18:24 [8]	17:50 [7]
Autonomic indexes	SNSi	1.5 [1.5]	0.2 [1.7] ^a^	0.9 [0.6]	0.6 [0.5] ^a^	17:27 [2]	16:30 [5]^c^
	PNSi	−1.09 [1.1]	0.42 [2.9] ^a^	0.7 [0.5]	0.5 [0.5]	04:46 [2]	02:38 [7] ^a^
	Stress Index (STi)	13.7 [7.2]	10.6 [7.4] ^a^	2.4 [2.1]	1.7 [2.2] ^b^	16:30 [6]	16:13 [6]

All values are presented as median [IQR]. Statistically significant differences in the Mann–Whitney test are indicated by superscripts a = *p* < 0.001, b = *p* < 0.01, c = *p* < 0.05. All values within acrophase are presented in hours and minutes (hh:mm) and in brackets are in hours (hh) to improve visualization. Other units as presented in the ‘Variable’ column. Blue: significant statistical differences. Red: variables that showed uniformity in CR.

**Table 4:** Effect of beta-blocker therapy on circadian parameters by variable.

	Parameter	β_BB	*p*_BB	q_BB_FDR	β_CR	*p*_CR	β_Age	*p*_Age	β_Sex	*p*_Sex
REC	Amplitude	2.22	0.01	0.415	0.78	0.28	0.06	0.01	0.73	0.24
PNN	MESOR	−14.32	0.0002	0.0045	18.61	0.001	0.08	0.45	−0.69	0.78
ShannE	MESOR	0.19	0.001	0.010	−0.15	0.002	−0.001	0.42	−0.04	0.32
REC	MESOR	7.67	0.001	0.010	−3.19	0.09	0.07	0.25	−1.67	0.29
SD2	MESOR	−22.25	0.005	0.035	28.24	0.01	0.12	0.60	−5.24	0.33
SDNN	MESOR	−24.41	0.007	0.036	34.96	0.01	0.38	0.14	−4.55	0.45
DET	MESOR	0.79	0.009	0.039	−1.005	0.0001	−0.01	0.09	−0.28	0.16
RMSSD	MESOR	−36.23	0.012	0.040	60.11	0.01	1.01	0.01	−5.28	0.59
SD1	MESOR	−25.66	0.012	0.040	42.57	0.01	0.71	0.01	−3.73	0.59

Multivariable linear models evaluating the effect of beta-blockers (BB) on circadian parameters in the whole cohort, adjusted for group (healthy/CR), age, and sex. Only variables showing significant BB effects are displayed. MESOR and amplitude were modeled using linear regression. Significance was assessed using *p*-values and false discovery rate (FDR) correction.

## Data Availability

The data presented in this study are available from the corresponding author upon reasonable request. The data are not publicly available due to privacy and ethical restrictions, in accordance with the data-use agreement and institutional regulations of the Instituto del Corazón de Bucaramanga.

## Supplementary Materials

The following supporting information can be downloaded at: https://www.mdpi.com/article/10.3390/biomedicines14051054/s1, Table S1: Uniformity tests (Repeated measures ANOVA and Rayleigh test); Table S2: Clinical characteristics of CR patients; Table S3: Watson–Williams circular test for acrophases; Table S4: Group comparison after excluding subjects with higher than 10% non-NN intervals.

## Abbreviations

The following abbreviations are used in this manuscript:

ANS	Autonomic Nervous System
BMI	Body Mass Index
CR	Cardiac Remodeling
ECG	Electrocardiogram
HF	Heart Failure
HFimpEF	Heart Failure with Improved Ejection Fraction
HFpEF	Heart Failure with Preserved Ejection Fraction
HFrEF	Heart Failure with Reduced Ejection Fraction
HFn	Normalized High Frequency
HFab	Absolute High Frequency
HR	Heart Rate
HRV	Heart Rate Variability
LFab	Low Frequency
LFn	Normalized Low Frequency
MESOR	Midline Estimating Statistic of Rhythm
NN	Normal-to-Normal (RR) intervals
PNSi	Parasympathetic Nervous System Index
RAAS	Renin–Angiotensin–Aldosterone System
RMSSD	Root Mean Square of Successive Differences
SDNN	Standard Deviation of NN intervals
SNSi	Sympathetic Nervous System Index
STi	Stress Index
VLF	Very Low Frequency
AmpEN	Amplitude Entropy
SampEn	Sample Entropy
ShannonE	Shannon Entropy
CorrDim	Correlation Dimension
REC	Recurrence
DET	Determinism
SD1	Poincaré short-term variability
SD2	Poincaré long-term variability
DFA	Detrended Fluctuation Analysis
SCN	Suprachiasmatic Nucleus
Φ	Acrophase
