# Supplementary material for: Circadian Activity Disruption in Cardiac Remodeling Patients Underlies Autonomic Dysfunction in Heart Failure
Source: Biomedicines. 2026 May 6;14(5):1054. doi: 10.3390/biomedicines14051054 (PMC13203849; doi:10.3390/biomedicines14051054)
Supplement: Supplementary file 1 [file biomedicines-14-01054-s001.zip › biomedicines-4195951-supplementary.pdf]

Supplementary material

Table S1. Uniformity tests (Repeated measures ANOVA and Rayleigh test). Non-uniformity for ANOVA: F > 1 p <0.001 and Rayleigh test.

**Analysis**	**Variable**	**ANOVA**	
		CR	Control
**Time domain(3)**	SDNN	$F_{23,86}$= 2.50, p<0.001	$F_{23,86}$= 2.61, p<0.001
	RMSSD	$F_{23,86}$= 2.80, p<0.001	$F_{23,86}$= 3.73, p<0.001
	pNN50	$F_{23,86}$= 2.89, p<0.001	$F_{23,86}$= 4.95, p<0.001
**Frequency domain(4)**	HF_n	$F_{23,86}$= 2.01, p=0.003	$F_{23,86}$= 4.88, p<0.001
	LF_n	$F_{23,86}$= 1.98, p=0.003	$F_{23,86}$= 4.84, p<0.001
	VLF	$F_{23,86}$= 0.86, p=0.649	$F_{23,86}$= 1.65, p=0.02
	LF/HF	$F_{23,86}$= 2.17, p=0.001	$F_{23,86}$= 19.07, p<0.001
**Non-linear(9)**	SD1	$F_{23,86}$= 2.82, p<0.001	$F_{23,86}$= 3.73, p<0.001
	SD2	$F_{23,86}$= 2.04, p=0.002	$F_{23,86}$= 1.97, p=0.03
	SD2/SD1	$F_{23,86}$= 1.00, p=0.457	$F_{23,86}$= 3.68, p<0.001
	SamPEN	$F_{23,50}$= 1.32, p=0.14	$F_{23,86}$= 3.11, p<0.001
	AmpEN	$F_{23,86}$= 1.94, p=0.004	$F_{23,86}$= 1.68, p=0.02
	Alf1	$F_{23,86}$= 1.14, p=0.289	$F_{23,86}$= 3.79, p<0.001
	Alf2	$F_{23,86}$= 1.73, p=0.01	$F_{23,86}$= 3.75, p<0.001
	CorrDim	$F_{23,86}$= 2.40, p<0.001	$F_{23,86}$= 1.76, p=0.01
	REC	$F_{23,86}$= 2.19, p<0.001	$F_{23,86}$= 1.10, p=0.32
	DET	$F_{23,86}$= 1.45, p=0.07	$F_{23,86}$= 2.12, p=0.001
	ShannE	$F_{23,50}$= 2.09, p=0.001	$F_{23,50}$= 1.21, p=0.223
**Autonomic indexes(3)**	SNS	$F_{23,86}$= 9.45, p<0.001	$F_{23,86}$= 7.14, p<0.0001
	PNS	$F_{23,86}$= 6.03, p<0.001	$F_{23,86}$= 11.72, p<0.0001
	Stress index	$F_{23,86}$= 2.68, p<0.001	$F_{23,86}$= 2.52, p<0.0001
	**Variable**	**Rayleigh**	
		**CR**	**Control**
**Time domain(3)**	**SDNN**	A: R= 0.15 p=0.12<br>Φ: R= 0.42 p=0<br>M: R= 0.09 p=0.42	A: R= 0.16 p=0.08<br>Φ: R= 0.37 p=0<br>M: R= 0.09 p=0.48
	**RMSSD**	A: R= 0.23 p=0.08	A: R= 0.10 p=0.38

		Φ: R= 0.34 p=0<br>M: R= 0.15 p=0.11	Φ: R= 0.43 p=0<br>M: R= 0.12 p=0.26
	**pNN50**	A: R= 0.22 p=0.01<br>Φ: R= 0.38 p=0<br>M: R= 0.11 p=0.35	A: R= 0.27 p=0.001<br>Φ: R= 0.46 p=0<br>M: R= 0.17 p=0.07
**Frequency domain(4)**	**HF_n**	A: R= 0.26 p=0.002<br>Φ: R= 0.25 p=0.03<br>M: R= 0.05 p=0.77	A: R= 0.11 p=0.31<br>Φ: R= 0.60 p=0<br>M: R= 0.14 p=0.17
	**LF_n**	A: R= 0.25 p=0.03<br>Φ: R= 0.25 p=0.03<br>M: R= 0.05 p=0.78	A: R= 0.11 p=0.32<br>Φ: R= 0.59 p=0<br>M: R= 0.14 p=0.16
	**VLF**	A: R= 0.11 p=0.31<br>Φ: R= 0.46 p=0<br>M: R= 0.05 p=0.76	A: R= 0.09 p=0.43<br>Φ: R= 0.38 p=0<br>M: R= 0.09 p=0.43
	**LF/HF**	A: R= 0.72 p=0<br>Φ: R= 0.26 p=0.002<br>M: R= 0.32 p<0.001	A: R= 0.45 p=0<br>Φ: R= 0.50 p=0<br>M: R= 0.26 p=0.002
**Non-linear(9)**	**SD1**	A: R= 0.11 p=0.33<br>Φ: R= 0.34 p=0<br>M: R= 0.07 p=0.62	A: R= 0.32 p<0.001<br>Φ: R= 0.43 p=0<br>M: R= 0.24 p=0.05
	**SD2**	A: R= 0.16 p=0.09<br>Φ: R= 0.42 p=0<br>M: R= 0.08 p=0.57	A: R= 0.04 p=0.81<br>Φ: R= 0.34 p=0<br>M: R= 0.07 p=0.65
	**SD2/SD1**	A: R= 0.97 p=0<br>Φ: R= 0.08 p=0.57<br>M: R= 0.8 p=0	A: R= 0.99 p=0<br>Φ: R= 0.5 p=0<br>M: R= 0.8 p=0
	**SamPEN**	A: R= 0.99 p=0<br>Φ: R= 0.13 p=0.18<br>M: R= 0.91 p=0	A: R= 0.95 p=0<br>Φ: R= 0.1 p=0.3<br>M: R= 0.82 p=0
	**AmpEN**	A: R= 0.99 p=0<br>Φ: R= 0.19 p=0.04<br>M: R= 0.98 p=0	A: R= 0.90 p=0<br>Φ: R= 0.12 p=0.5<br>M: R= 0.88 p=0
	**Alf1**	A: R= 0.99 p=0	A: R= 0.99 p=0

		Φ: R= 0.14 p=0.18<br>M: R= 0.95 p=0	Φ: R= 0.58 p=0<br>M: R= 0.97 p=0
	**Alf2**	A: R= 0.99 p=0<br>Φ: R= 0.98 p=0<br>M: R= 0.98 p=0	A: R= 0.99 p=0.08<br>Φ: R= 0.53 p=0<br>M: R= 0.99 p=0.48
	**CorrDim**	A: R= 0.91 p=0<br>Φ: R= 0.17 p=0.07<br>M: R= 0.69 p=0	A: R= 0.92 p=0<br>Φ: R= 0.35 p=0<br>M: R= 0.63 p=0
	**REC**	A: R= 0.02 p=0.94<br>Φ: R= 0.27 p=0.01<br>M: R= 0.11 p=0.33	A: R= 0.05 p=0.7<br>Φ: R= 0.26 p=0.021<br>M: R= 0.60 p=0.7
	**DET**	A: R= 0.87 p=0<br>Φ: R= 0.18 p=0.06<br>M: R= 0.44 p=0	A: R= 0.95 p=0<br>Φ: R= 0.51 p=0<br>M: R= 0.8 p=0
	**ShannE**	A: R= 0.99 p=0<br>Φ: R= 0.29 p<0.001<br>M: R= 0.94 p=0	A: R= 0.99 p=0<br>Φ: R= 0.29 p<0.001<br>M: R= 0.98 p=0
**Autonomic indexes(3)**	**SNS**	A: R= 0.87 p=0<br>Φ: R= 0.57 p=0<br>M: R= 0.40 p=0	A: R= 0.86 p=0<br>Φ: R= 0.69 p=0<br>M: R= 0.48 p=0
	**PNS**	A: R= 0.81 p=0<br>Φ: R= 0.53 p=0<br>M: R= 0.07 p=0.62	A: R= 0.92 p=0<br>Φ: R= 0.73 p=0<br>M: R= 0.48 p=0
	**Stress index**	A: R= 0.36 p=0<br>Φ: R= 0.42 p=0<br>M: R= 0.05 p=0.78	A: R= 0.40 p=0<br>Φ: R= 0.43 p=0<br>M: R= 0.07 p=0.62

Table S2. Clinical characteristics of CR patients.

Variable	Cardiac remodeling (n=86)
**Age (years: median-IQR)**	58-17
**Females (n: %)**	38-32.7%
**White ethnicity (n-%)**	70-81.3%
**BMI (median-IQR)**	24.22-2.32
**Self-reported smokers (n-%)**	10-11.6%
**Hypertension (n-%)**	43-50%
**Coronary Artery Disease (n-%)**	29-33.7%
**Stroke (n-%)**	5-5.81%
**Arrhythmias (n-%)**	39-45.34%
**Diabetes mellitus 2 (n-%)**	11-12.7%
**SAHOS (n-%)**	7-8.1%
**Heart Failure (n-%)**	33-38.37%
**Prescribed drugs**	61-70.93%
**Betablockers**	33-38.4%
**Amiodarone**	14-16.2%
**Calcium antagonists**	35-40.69%
**ARB**	10-11.62%
**ACEI**	50-58.13%
**Statin**	17-19.76%
**DOAC**	2-2.32%
**Warfarin**	50-58.13%
**AAS**	30-34.88%
**Clopidogrel**	20-23.25%
**Diuretic**	21-24.41%
**Echocardiogram (Mdn-IQR)**	
**Left ventricular end-diastolic diameter (mm)**	4.8-1.17
**Left ventricular end-systolic (mm)**	3.2-1.2
**Interventricular septal thickness (mm)**	1.2-0.2
	216.17-112.3

**Left ventricular mass index (g/m²)**	0.48-0.11
**Posterior wall thickness (mm)**	55-14.7
**Left ventricular ejection fraction (%)**	4.2-1.1
**Left atrial diameter (mm)**	3.3-0.6
**HF classification by EF**	
**HFrEF**	18–21%
**HFimpEF**	10–11.6%
**HFpEF**	58–67.4%

Table S3. Watson-Williams circular test for acrophases

Variable	N_Healthy	N_CR	MeanΦ_Healthy	MeanΦ_CR	F_WW	p_WW
PNSi	86	86	2.53	1.71	2.91	0.090
SNSi	86	86	1.11	23.10	8.83	0.003
Sti	86	86	0.41	22.79	5.00	0.027
SDNN	86	86	1.44	0.76	1.09	0.297
MinHR	86	86	1.39	0.14	3.88	0.051
MaxHR	86	86	1.36	23.25	14.78	0.000
RMSSD	86	86	2.27	0.35	9.62	0.002
PNN	86	86	2.46	1.92	0.75	0.387
VLFab	86	86	0.64	0.56	0.01	0.916
LFfab	86	86	1.16	0.51	0.93	0.336
Hfab	86	86	1.97	1.17	1.85	0.176
LFn	86	86	0.98	0.30	1.23	0.268
HFn	86	86	1.80	0.64	4.40	0.038
TPw	86	86	1.20	1.25	0.00	0.944
LFHF	86	86	0.76	0.40	0.32	0.572
SD1	86	86	2.27	0.34	9.64	0.002
SD2	86	86	1.25	0.33	2.01	0.158
SD2SD1	86	86	1.09	0.40	1.22	0.271
AmpEN	86	86	23.31	23.78	0.38	0.541
SampEN	86	86	0.79	23.56	4.37	0.038
Alf1	86	86	0.87	0.33	0.66	0.417
Alf2	86	86	0.44	0.05	0.49	0.484
CorrDimD2	86	86	2.07	1.66	0.43	0.515
REC	86	86	0.31	0.00	0.24	0.626
DET	86	86	23.82	0.53	1.17	0.282
ShannE	86	86	0.08	23.91	0.07	0.789

Table S4. Group comparison after excluding subjects with high non-NN intervals

Values are expressed as mean ± SD. Comparisons between Healthy and CR groups of MESOR were performed using the Mann–Whitney U test.

Variable	Healthy_mean	Healthy_sd	CR_mean	CR_sd	p_value
**Alf1**	1.249131638	0.232325313	0.878493	0.310148	1.14E-13
**SD2SD1**	2.535909366	0.673549394	1.628774	0.661565	4.59E-13
**LFn**	71.54897219	12.20441818	49.28131	20.138939	7.36E-13
**HFn**	28.39430804	12.13753015	50.54344	20.010503	7.55E-13
**LFHF**	4.70465189	2.52162226	2.250752	2.082747	7.43E-11
**PNSi**	-0.932494622	0.822966402	0.589347	2.2269353	7.33E-09
**RMSSD**	27.92921042	16.54575583	64.98205	70.301192	4.88E-06
**SD1**	19.77854296	11.71822037	46.02067	49.791132	4.88E-06
**Hfab**	337.6431912	368.8143336	2136.357	4871.8459	5.63E-05
**SNSi**	1.618078101	1.355812354	0.886745	2.0290488	5.97E-05
**MaxHR**	90.85891279	10.05018846	83.50637	17.050143	6.14E-05
**AmpEN**	1.097491764	0.067178026	1.017089	0.1699668	0.000902
**Alf2**	0.489703391	0.085640786	0.438427	0.1642796	0.00175
**MinHR**	67.24300921	8.768335285	62.53893	11.96021	0.002302
**Sti**	14.79421904	5.177619663	13.68015	9.0293552	0.00515
**PNN**	7.340039244	7.343893967	15.68674	18.375987	0.005427
**SDNN**	32.86972297	11.95105898	50.50755	43.371983	0.024979
**DET**	98.12543711	0.671032534	97.60728	1.5166615	0.046114
**CorrDimD2**	1.227497868	0.900539623	1.030345	0.9461313	0.089862
**TPw**	1225.878702	846.2772232	4922.369	12316.079	0.158109
**LFfab**	755.7009439	563.5119722	2126.597	5899.5793	0.180149
**REC**	35.52032563	7.02073675	38.07496	12.577609	0.238944
**SD2**	41.19519849	13.95917175	51.99336	37.88036	0.305301
**VLFab**	131.8613932	78.1294122	654.1271	3146.9441	0.675564
**SampEN**	1.45533813	0.187236092	1.36178	0.4068721	0.819065
**ShannE**	3.224162403	0.166927052	3.206226	0.2935663	0.906927
